# Endothelial–Vascular Smooth Muscle Cells Interactions in Atherosclerosis

**DOI:** 10.3389/fcvm.2018.00151

**Published:** 2018-10-23

**Authors:** Manna Li, Ming Qian, Kathy Kyler, Jian Xu

**Affiliations:** ^1^Department of Medicine, Harold Hamm Diabetes Center, University of Oklahoma Health Sciences Center, Oklahoma, OK, United States; ^2^Office of Research Administration, University of Oklahoma Health Sciences Center, Oklahoma, OK, United States

**Keywords:** atherosclerosis, cardiovascular disease, cell interactions, endothelial cells, vascular smooth muscle cells

## Abstract

Atherosclerosis is a chronic progressive inflammatory process that can eventually lead to cardiovascular disease (CVD). Despite available treatment, the prevalence of atherosclerotic CVD, which has become the leading cause of death worldwide, persists. Identification of new mechanisms of atherogenesis are highly needed in order to develop an effective therapeutic treatment. The blood vessels contain two primary major cell types: endothelial cells (EC) and vascular smooth muscle cells (VSMC). Each of these performs an essential function in sustaining vascular homeostasis. EC-VSMC communication is essential not only to development, but also to the homeostasis of mature blood vessels. Aberrant EC-VSMC interaction could promote atherogenesis. Identification of the mode of EC-VSMC crosstalk that regulates vascular functionality and sustains homeostasis may offer strategic insights for prevention and treatment of atherosclerotic CVD. Here we will review the molecular mechanisms underlying the interplay between EC and VSMC that could contribute to atherosclerosis. We also highlight open questions for future research directions.

## Introduction

Atherosclerosis, or hardening of the atrial blood vessel wall, is a chronic progressive inflammatory disorder. The disorder presents with coronary artery disease, carotid artery disease, peripheral artery disease, or combined, cardiovascular disease (CVD) (1). With life-threatening complications, including myocardial infarction and stroke, CVD is the leading cause of death worldwide. Despite available treatments, CVD prevalence continues, suggesting an urgent need to identify the pathogenic molecular mechanisms and develop effective therapeutic approaches. Blood vessel walls are comprised primarily of endothelial cells (EC) and vascular smooth muscle cells (VSMC). Each cell type has an important role in vascular homeostasis. Interaction between these two major cell types is fundamental not only to the development and formation of the vasculature, but also to the function of mature vasculature (2), such as maintaining vessel tone in mature vessels. Their communication is critical for repair and remodeling associated with blood vessel growth. A Compendium on Atherosclerosis (3) recently provided comprehensive reviews on the roles of ECs (4) and VSMCs (5) in the pathological progression of atherogenesis. However, the modes and molecular mechanisms of the EC-VSMC conversation that causes atherosclerosis are less known. Identification of the pathways underlying EC-VSMC interaction-mediated vascular homeostasis in the course of atherogenesis can offer strategic insights for the prevention and treatment of atherosclerotic CVD. While general functions of individual ECs and VSMCs have been extensively reviewed elsewhere, the present review summarizes the emerging evidence that connects physical (direct) and biochemical (indirect) crosstalk between ECs and VSMCs to atherogenesis, and highlights the unanswered questions that merit future investigations.

## Roles of EC and VSMC in atherosclerosis

Atherosclerotic lesion formation is a complex process, with initiation and progression dependent on a localized inflammatory response that facilitates changes in the vessel wall (6, 7). Fatty streaks in arterial walls gradually develop into atheroma and characteristic plaques. The acute rupture of these atheromatous plaques causes local thrombosis, leading to partial or total occlusion of the affected artery. The clinical consequences of these plaques depend on their site and the degree and speed of vessel occlusion. The disease has a latency of many years, and frequently coexists in more than one vascular bed. Its major clinical manifestations include ischemic heart disease, ischemic stroke, and peripheral arterial disease.

Abnormal heterotypic cell communication can cause vascular defects (4). A major piece of evidence supporting this notion is that endothelial dysfunction, a well-defined pathological state of the endothelium, underlies vascular impairment in atherosclerosis (8, 9), hypertension, hypercholesterolemia, and diabetes (10). A detectable change in the vascular reactivity and composition of the vascular wall is a common feature of these diseases. It is widely accepted that the effects of endothelial dysfunction on VSMCs are reduction of NO bioavailability and/or augmentation of vasoactive constrictors released from the endothelium (8, 9). Endothelial dysfunction has been positively associated with the pathology of metabolic disorders and the related vascular complications (10, 11). VSMCs, another major type of vascular cell, play a crucial role in the initiation and development of atherosclerosis (6). Mechanistically, normal and controlled VSMC proliferation is beneficial in atherogenesis, while dysregulated VSMC proliferation contributes to plaque formation and aberrant inflammation (5, 12). Thus, endothelial dysfunction contributes to impairment of NO-dependent vasodilatation, cellular glucose uptake, enhanced oxidative stress, and inflammation, leading eventually to atherosclerosis (1, 13–16).

## Modes of EC-VSMC interactions in atherosclerosis

The individual functions of ECs and VSMCs are dependent on their proper interaction, which is fundamental to the formation and function of the vasculature (2). The early interactions begin at embryogenesis when the blood vessels are forming (2, 17). The intimate EC-VSMC interaction may also determine the outcome of vascular homeostasis under diseased conditions, including atherosclerosis. Great progress has been made in understanding EC-conveyed signals to SMC regulating vascular tone and the basic interplay that occurs during vessel assembly. However, modes of EC-VSMC communication in adults can be very different from those in developing humans. For vessel assembly, the proliferation and migration of adult EC-VSMC cells are less dynamic. Moreover, physical interactions of EC-VSMC might be blocked by the basement membrane and the internal elastic lamina in mature blood vessels. ECs and VSMCs have evolved various modes of interaction to regulate vascular function and sustain homeostasis. Although it remains largely unclear how defects in EC-VSMC interaction could lead to atherosclerosis, an overview of the mode of EC-VSMC interaction is timely and will help to identify key outstanding problems.

## EC-VSMC interaction *via* direct contact

EC-VSMC interaction *via* direct contact, which has contributed to arterial-venous identity, vascular tip cell specification and sprouting, and VSMC differentiation in vascular development, occurs in embryonic growth (2). In adult vasculature, junctional molecules, such as intercellular adhesion molecules, mediate most of the direct contact between vascular cells (18) N-cadherin, which was believed to mediate EC-VSMC physical adhesion, was found in layers of ECs and VSMCs beneath the internal elastic membrane in adult vasculature (19). Connexins are the next regulator of EC-VSMC interaction in adult blood vessels (20). Connexin 43 post-translational modification by nitrosylation (21) and phosphorylation (22), respectively, alters vascular reactivity. Like ECs, VSMCs also express intercellular adhesion molecule 1 (ICAM-1) and vascular cell adhesion molecule 1 (VCAM-1) in atherosclerosis (23), restenosis (24), and transplant vasculopathy (25). There are reports of direct communications between ECs and VSMCs that affect vasculature formation. *In vitro* data suggested that EC-expressed Jagged1 could interact with NOTCH3 on neighboring SMCs, which activated NTOCH signaling, promoting more NTOCH3 expression in the SMCs (26). Another example of contact-dependent interplay involves Ephrin receptor tyrosine kinases (Eph), which are activated by binding to ephrin ligands that are linked to the cell membrane *via* glycosylphosphatidylinisotol anchor (ephrin A) or *via* a transmembrane domain (ephrin B) (27). Among well-studied receptor-ligand pairs (28), a reciprocal interaction of EphB4 and ephrin-B2 from both ECs and VSMCs is required in growing blood vessels (29). Both ephrin and receptors have been detected in atherosclerotic plaques (30, 31), indicating a strong association of these proteins with the inflammatory outcome in atherosclerosis (32). A causal role of the direct contact in atherogenesis has yet to be established; however, data for a role of indirect EC-VSMC interaction are emerging.

## EC-VSMC interaction *via* eNOS-derived NO

While developmental signals are required by mature vessels for basal function, they rely on further interactions regulating such vascular functions as vascular tone and blood pressure (33). These signals operate by employing endothelium-derived hyperpolarizing factor (EDHF) (e.g., eNOS-derived NO) and gap junctions that couple EC-VSMC. Mechanistically, EDHF-induced hyperpolarizing current spreads quickly, leading to vasodilatation and thereby increasing blood flow, while small molecules (e.g., Ca^2+^) also coordinate changes in diameter and modulate vascular responses. EC-derived NO has also been reported to change flow-dependent vascular remodeling by negatively regulating the platelet-derived growth factor (PDGF) pathway (34). Moreover, increased myoendothelial junctions could be a way of enhancing the interaction, as observed in caudal arteries of spontaneously hypertensive rats (35). In contrast, pharmacological blocking of myoendothelial GAP-junction impaired EC-induced contraction (36). These data suggest EC-mediated SMC hyperpolarization as a mode of EC-VSMC conversation (37, 38).

## EC-VSMC interaction *via* the extracellular matrix (ECM)

A primary feature of atherosclerotic plaques is a transition state of VSMCs, which become proliferative and secrete excess ECM to build up the plaque lesion (6). The ECM was traditionally regarded as a cellular scaffold or foundation to maintain the mechanical properties of blood vessels. It is now known as a source of signaling mediators (39). Alterations in the ECM have structural implications and signaling changes that disrupt EC-VSMC interactions. Both ECs and SMCs synthesize and secrete ECM, which is a complex mixture of components derived from ECs and VSMCs (39, 40), and could influence the function of neighboring cells (41). Indeed, interventional angioplasty to remove diseased plaques may induce EC denudation, damage, and further dysfunction, attributable to the loss of the suppressive effects on VSMC proliferation, thereby causing restenosis (42).

## EC-VSMC interaction *via* extracellular vesicles

Extracellular vesicles (EVs) are phospholipid bilayer-enclosed membrane sacs that emerged as a mechanism regulating cell-cell communication (43). EVs include exosomes, microparticles, and apoptotic bodies, which carry biomolecules, such as proteins, DNA, mRNA, and noncoding RNA (44). Under physiological conditions, ECs constitutively secrete low concentrations of EVs into the circulation. However, endothelial EV levels increase under various diseases conditions involving endothelial injury or dysfunction (45). EC-derived EVs contain proteins with emerging roles in atherogenesis (43). EVs have been reported to function in post-plaque rupture responses, which promote tissue factor, a rate-limiting enzyme, to initiate the coagulation cascade. Both ECs (46) and VSMCs (47) can release TF-loaded VEs; however, it remains unknown how EC and VSMC talk to each other to control the proper release of the same factor. Notably, one of the cargoes carried by EC-derived EVs is miRNA, a discussion of which follows below.

## EC-VSMC interaction *via* MicroRNAs (MiRNAs)

MiRNAs are evolutionarily conserved and noncoding small RNAs. miRNAs are secreted from cells and can be picked up by other cells (48). MiRNAs function as important regulators and fine-tuners of a range of pathophysiological cellular effects and molecular signaling pathways involved in atherosclerosis (49). Early studies demonstrated that miRNAs mediate atheroprotective communication between EC-VSMC (50). A recent study showed that ECs could inform VSMCs to proliferate *via* a direct secretion of miR-126 from ECs to VSMCs (51), which augments VSMC turnover and worsens atherosclerosis. In line with these findings, atheroprotective shear stress blocked miR-126 release (51). A similar atheroprotective effect was observed when EC-derived miR-143/145 were transferred to VSMCs through an EV-mediated pathway (50). In this regard, miRNAs function similarly to secreted proteins and peptides, which have been considered as major regulators for communication among vascular cells.

## EC-VSMC interaction *via* other factors affecting SMC cell turnover

EC-VSMC dialogue can alter developmental signaling pathways in mature blood vessels. Hemodynamic force stimulates ECs to produce heparin sulfate proteoglycans, which promote vascular growth and hypertrophy (52). This was achieved by enhancing the VSMC response to growth signals from transforming growth factor beta (TGF-β) (52). Manipulation of EC can promote excessive VSMC turnover in plaque formation. The EC injury-activated PDGF signaling pathway is associated with VSMC proliferation and ECM synthesis (53). Similarly, loss of EC-expressed Apelin, an endogenous ligand for G protein–coupled receptors, causes defects in vascular maturation and VSMC recruitment (54), suggesting an overlap with the PDGF pathway. In contrast, EC-FGF receptor signaling accelerates atherosclerosis (55), whereas EC-overexpression of FasL decreases atherosclerosis in *ApoE*^−/−^ mice (56). Homocysteine activates VSMCs by DNA demethylation of PDGF in ECs (57). Another atheroprotective mode of EC-VSMC interaction is supported by evidence showing EC-induced suppression of SMC proliferation and, thus, vascular injury. These effects have been accomplished with blood vessel re-endothelialization by blocking cell migration (58) and restenosis (59), elevating peroxiredoxin activity (60), and inducing VSMC apoptosis (61), respectively. These data further support the therapeutic potential of promoting EC regrowth after tissue damage.

Wnt-signaling is involved in many aspects of the atherogenesis (62–64) including EC dysfunction (65), macrophage activation (66), and VSCM proliferation (67). For example, canonical Wnt/β-catenin pathway regulates VSMC proliferation and survival *via* a crosstalk between the Wnt cascade and NF-κB signaling, mediated through β-TrCP1, an E3-ligase (68). Wnt-signaling dependent EC-VSMC interaction, however, is less known. Recent studies showed that EC-derived non-canonical Wnt ligand regulated vascular formation in an autocrine manner (69). In line with these results, enhancement of Wnt-signaling in ECs through R-spondin3 was required for vascular stability during vasculature remodeling (70). Given the EC-VSMC interplay in atherogenesis, components of the Wnt signaling cascade may represent novel targets for atherosclerosis (71).

In an analogy to transdifferentiation of VSMCs to macrophage-like cells during atherogenesis (72), ECs have been shown to have certain plasticity through interacting with ECM and/or cues from supporting cells. Indeed, transdifferentiation of mature vascular ECs has been detected in pulmonary hypertension, which plays an important role in pulmonary arterial remodeling (73). This likely happens due to downregulation of EC-cadherin (74) or regulation by myocardin in hypoxia-induced pulmonary vascular remodeling (75). However, the causal role of EC-derived VSMC in atherogenesis, if any, remains poorly understood (5).

## EC-VSMC two-way interaction

It is widely accepted that EC dysfunction is a leading cause of atherosclerosis (76). The resultant dysfunctional VSMCs contribute to atherogenesis (5). Specifically, loss of endothelial cell function elicits abnormal expression of adhesion proteins that recruit leukocytes from the blood into vascular tissue, wherein these cells promote VSMC-mediated vascular wall remodeling. As such, atherosclerosis is characterized by chronic vascular wall inflammation, progressive narrowing of the vessel lumen, and eventual plaque formation. However, EC-VSMC communication is not unidirectional from blood into the vascular wall in atherogenesis, or simply from EC to VSMC. Changes that occur in VSMCs may ultimately affect the other side of the conversation. In a mouse model of thoracic aortic aneurysm, elevated endoplasmic reticulum stress in VSMC stimulated the release of EVs, which contributed to EC apoptosis and the infiltration of inflammatory cells (77). VSMC-secreted ECM can buffer the high-pressure load of circulating blood, which prevents physical EC permeability in large vessels (78). Given the critical role of the two major cell types in atherogenesis, in-depth studies of the VSMC-derived impacts on ECs in atherosclerosis, a less-investigated area, should be encouraged.

## EC-VSMC interaction: genetic evidence

Defects in EC-VSMC interactions cause certain genetic diseases. Cerebral autosomal dominant arteriopathy with subcortical infarcts and leukoencephalopathy (CADASIL) is an inherited disease caused by *NOTCH3* mutations that lead to vascular dementia and stroke due to SMC degeneration in small arteries (79). Patients with CADASIL exhibit endothelial dysfunction (80). Animal models of CADASIL exhibit disruptions in vascular tone and increased incidence of ischemic stroke (81, 82). In addition, patients with Marfan syndrome have defects in the gene encoding ECM protein fibrillin-1, which cause structural abnormalities of the vessel wall, likely due to elevated TGF-signaling (83, 84). Endothelium-dependent vasomotor dysfunction was found in the small arteries of a mouse model of Marfan syndrome, suggesting defects in heterotypic cell communication (85). Endoglin is a TGF-β receptor for the TGF-mediated signaling pathway and is highly expressed in EC (86). *Endoglin* mutations cause hereditary hemorrhagic telangiectasia (HHT), which is an autosomal-dominant disorder. Patients with HHT manifest with dilations of the vascular lumen (87) and thinning of blood vessel wall (88), which lead to arteriovenous malformations and hemorrhage (89). Mutations in other mediators of TGF-β signaling could induce HHT (90). *Endoglin*-KO mice present with defects in EC-dependent SMC recruitment (91). Interestingly, heterozygous *endoglin* mutation has impaired NO-dependent reactivity (92), suggesting an additional function to maintain vascular tone in mature vessels.

## Outstanding questions and future directions

**Figure 1:**
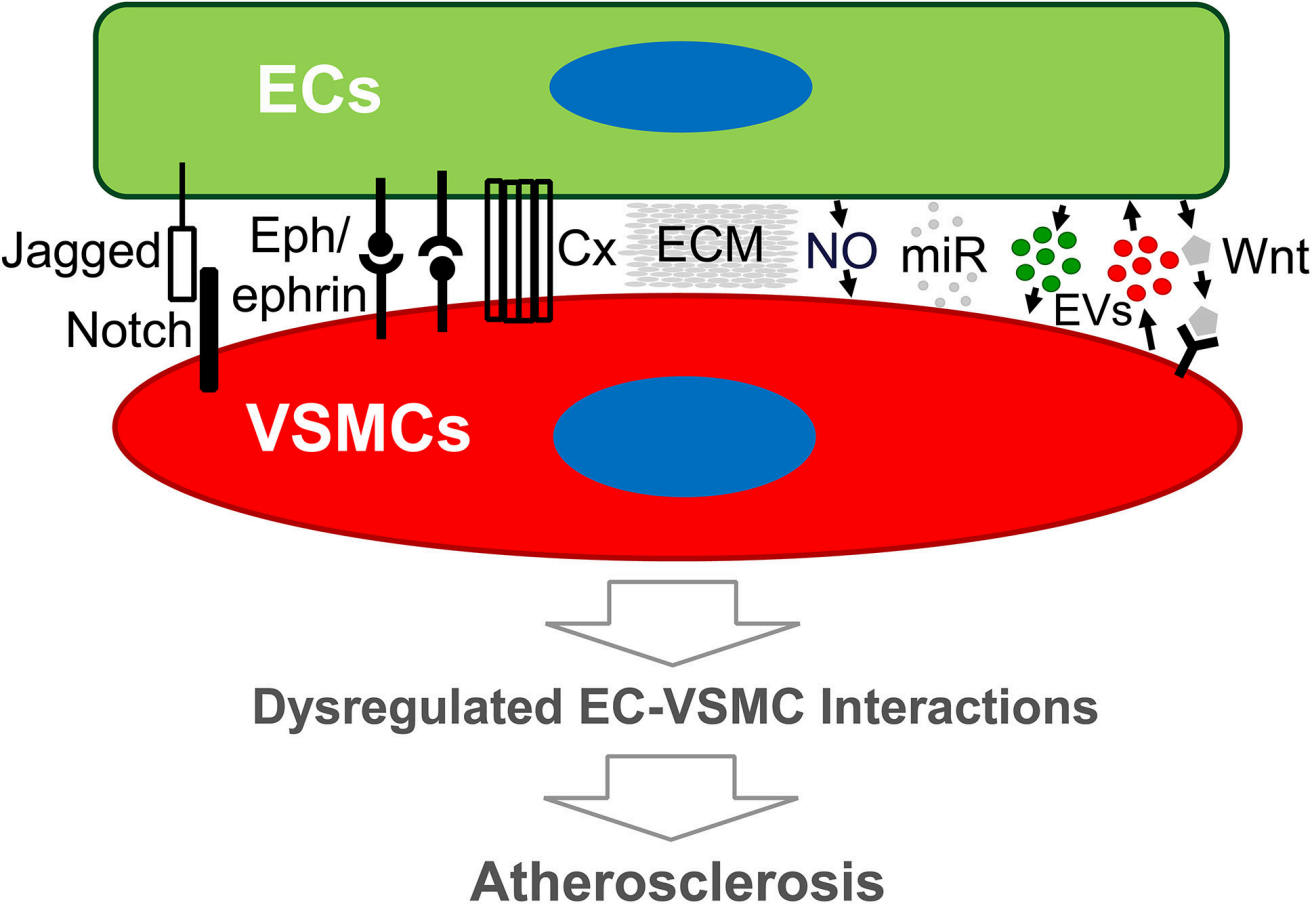
Schematic diagram of modes of endothelial cell-vascular smooth muscle cell interaction that may lead to atherosclerosis. Emerging evidence demonstrates that both direct and indirect interplay between ECs and VSMCs are functional. Direct EC-VSMC crosstalk involves physical contact through cell surface proteins, such as Connexin, Eph/ephrins, and Jagged/Notch3. Indirect EC-VSMC dialogue is biochemical interaction mediated by cell-released or secreted factors (e.g., EDHF, EVs, miRNA) and matrix (ECM). The outcome of the dialogue is expected to alter EC and/or VSMC functions that promote atherogenesis. Cx, Connexin, or other junction proteins; ECM, extracellular matrix; ECs, endothelial cells; EVs, extracellular vesicles; miR, micro-RNA; VSMCs, vascular smooth muscle cells; Wnt, Wnt ligand proteins.

Our understanding of atherogenesis has progressed significantly. The endothelium is recognized and implicated in the regulation of physiologic and pathologic processes *via* its signals and metabolic cues (93, 94) to their residing organs in development and function. Loss of endothelial function thus contributes to CVD (95, 96). This review focuses on EC-VSMC interaction-promoted atherogenesis, a less-explored, but potentially important, field. The existence of genetic disorders due to EC-VSMC interaction defects indicates the clinical significance of the modes of interplay. The reviewed pathways that ECs and VSMCs use to communicate in vascular functionality support an essential role for their interaction in atherosclerotic plaque formation (Figure 1). There are other major cell types in different stages of atherogenesis. The modes of interaction may well apply to the dialogue of ECs with other cell types in atherogenesis. Since a selected pathway or target may have opposing effects in different cell types, a promising therapeutic target would promote (net) beneficial outcomes in multiple cell types. Outstanding questions that warrant future exploration are listed below.

Endothelial dysfunction ultimately leads to atherosclerotic CVD. Treatment of endothelial dysfunction has focused mainly on reducing known CVD risk factors, because this approach could be associated with improved vascular endothelial functions (97). However, treatment specifically targeting the EC-dependent mechanism is not available. Can these drugs modulate the crosstalk between ECs and VSMC, and translate to the prevention and treatment of atherosclerosis? Emerging discoveries, including EC-mediated signaling (98), EC metabolism (99, 100), EC-mediated re-endothelialization (as seen in the treatment of stroke) (101), EC-regulated blood flow sensor function (102), EC-induced metabolic changes (103), and EC-vascular integrity, may be linked to the EC-VSMC crosstalk reviewed here (104–107). Could a better and/or more effective target be identified based on EC-centered mechanisms for atherosclerosis?

There are challenges to determining an authentic EC-VSMC interplay that causes vascular injury. The disruptions in signaling between ECs and VSMCs are difficult to precisely define, due to the contribution of other cell types, e.g., inflammatory cells, monocytes, and lymphocytes. An array of approaches from various perspectives has been reported, e.g., using a co-culture system to identify contributing cell type (108). using endothelial dysfunction as an early predictor of vascular cell conversation (109), text mining to identify genes associated with atherogenesis (110), and further classifying sub-population(s) of SMCs linking to their specificity (111). Would systematic consideration and/or application of these approaches be a better way to identify a causal role of EC-VSMC interaction in vascular injury?

Vascular cell communication confers on the blood vessel wall the ability to act as a functional entity. In addition to ECs and VSMCs, there are other major cell types in different stages of atherogenesis. The modes of interaction may well apply to the dialogue of ECs with other cell types, such as effector macrophages (112) and vascular first responder platelets (113), which significantly contribute to atherogenesis. Macrophages are the major and important type of cell that determines the progression of atherosclerosis by interplay with both ECs (4) and VSMCs (5). How is EC-VSMC signaling integrated with macrophages to determine the fate of atherosclerosis?

To date, therapies in the atherosclerosis field have mainly focused on drugs that control blood lipids (e.g., statins), which fail to significantly reduce disease prevalence. Anti-inflammatory strategies targeting macrophages and other immune cells remain unproven. Can we shift the paradigm to identify the factors and mechanisms that can promote beneficial vascular cell interactions, such as those between EC-VSMC, which can either enhance or replace current conventional anti-atherosclerotic therapies?

It has been recognized that VSMCs of different embryological origin may undergo specific processes at different stages and in different regions of the plaque during atherogenesis (5). These processes are associated with VSMC phenotypic switching, cell proliferation, migration, cell death, and cell senescence. What is the role and mechanism of ECs in these processes that eventually lead to atherosclerosis?

The impact of sex and gender differences has been widely described in cardiovascular diseases, including atherosclerosis (114–116). Although work with most available animal models (117) cannot address a sex-specific impact in atherogenesis [e.g., more plaque erosion in younger women (118)], emerging evidence has shown that sex affects cells that are involved in atherogenesis in humans (119). What is the role and mechanism of sex and gender differences in the EC-VSMC interaction that contributes to atherosclerosis?

In conclusion, atherosclerosis is a chronic arterial disease and a leading cause of vascular death. Our deeper understanding of the defects in EC-VSMC interaction that induce atherosclerosis may allow us to design proper targets for the treatment and prevention of atherosclerotic CVD.

## Author contributions

JX contributed to the conception. ML, MQ, KK, and JX wrote the article.

### Conflict of interest statement

The authors declare that the research was conducted in the absence of any commercial or financial relationships that could be construed as a potential conflict of interest.

## References

[1] Hopkins PN. Molecular biology of atherosclerosis. Physiol Rev. (2013) 93:1317–542. 10.1152/physrev.00004.2012 23899566

[2] Lilly B. We have contact: endothelial cell-smooth muscle cell interactions. Physiology(Bethesda) (2014) 29:234–41. 10.1152/physiol.00047.2013 24985327

[3] Libby P Bornfeldt KE Tall AR. Atherosclerosis: successes, surprises, and future challenges. Circ Res. (2016) 118:531–4. 10.1161/CIRCRESAHA.116.308334 26892955 PMC4762065

[4] Gimbrone MA Jr Garcia-Cardena G. Endothelial cell dysfunction and the pathobiology of atherosclerosis. Circ Res. (2016) 118:620–36. 10.1161/CIRCRESAHA.115.306301 26892962 PMC4762052

[5] Bennett MR Sinha S Owens GK. Vascular smooth muscle cells in atherosclerosis. Circ Res. (2016) 118:692–702. 10.1161/CIRCRESAHA.115.306361 26892967 PMC4762053

[6] Doran AC Meller N McNamara CA. Role of smooth muscle cells in the initiation and early progression of atherosclerosis. Arterioscler Thromb Vasc Biol. (2008) 28:812–9. 10.1161/ATVBAHA.107.159327 18276911 PMC2734458

[7] Lusis AJ. Atherosclerosis. Nature (2000) 407:233–41. 10.1038/35025203 11001066 PMC2826222

[8] Hirase T Node K. Endothelial dysfunction as a cellular mechanism for vascular failure. Am J Physiol Heart Circ Physiol. (2012) 302:H499–505. 10.1152/ajpheart.00325.2011 22081698

[9] Mombouli JV and Vanhoutte PM. Endothelial dysfunction: from physiology to therapy. J Mol Cell Cardiol. (1999) 31:61–74. 10.1006/jmcc.1998.0844 10072716

[10] Xu J Zou MH. Molecular insights and therapeutic targets for diabetic endothelial dysfunction. Circulation (2009) 120:1266–86. 10.1161/CIRCULATIONAHA.108.835223 19786641 PMC2910587

[11] Rask-Madsen C King GL. Vascular complications of diabetes: mechanisms of injury and protective factors. Cell Metab. (2013) 17:20–33. 10.1016/j.cmet.2012.11.012 23312281 PMC3546345

[12] Weber C Noels H. Atherosclerosis: current pathogenesis and therapeutic options. Nat Med. (2011) 17:1410–22. 10.1038/nm.2538 22064431

[13] Tabas I. 2016 Russell Ross memorial lecture in vascular biology: molecular-cellular mechanisms in the progression of atherosclerosis. Arterioscler Thromb Vasc Biol. (2017) 37:183–9. 10.1161/ATVBAHA.116.308036 27979856 PMC5269511

[14] Linden F Domschke G Erbel C Akhavanpoor M Katus HA Gleissner CA. Inflammatory therapeutic targets in coronary atherosclerosis-from molecular biology to clinical application. Front Physiol. (2014) 5:455 10.3389/fphys.2014.00455 25484870 PMC4240064

[15] Bornfeldt KE. 2013 Russell Ross memorial lecture in vascular biology: cellular and molecular mechanisms of diabetes mellitus-accelerated atherosclerosis. Arterioscler Thromb Vasc Biol. (2014) 34:705–14. 10.1161/ATVBAHA.113.301928 24665124 PMC3967130

[16] Phinikaridou A Andia ME Shah AM Botnar RM. Advances in molecular imaging of atherosclerosis and myocardial infarction: shedding new light on *in vivo* cardiovascular biology. Am J Physiol Heart Circ Physiol. (2012) 303:H1397–410. 10.1152/ajpheart.00583.2012 23064836 PMC3532533

[17] Marcelo KL Goldie LC Hirschi KK. Regulation of endothelial cell differentiation and specification. Circ Res. (2013) 112:1272–87. 10.1161/CIRCRESAHA.113.300506 23620236 PMC3768127

[18] Liebner S Cavallaro U Dejana E. The multiple languages of endothelial cell-to-cell communication. Arterioscler Thromb Vasc Biol. (2006) 26:1431–8. 10.1161/01.ATV.0000218510.04541.5e 16556854

[19] Gilbertson-Beadling SK Fisher CA potential role for N-cadherin in mediating endothelial cell-smooth muscle cell interactions in the rat vasculature Lab Invest. (1993) 69:203–9. 8350598

[20] Isakson BE Damon DN Day KH Liao Y Duling BR. Connexin40 and connexin43 in mouse aortic endothelium: evidence for coordinated regulation. Am J Physiol Heart Circ Physiol. (2006) 290:H1199–205. 10.1152/ajpheart.00945.2005 16284228

[21] Straub AC Billaud M Johnstone SR Best AK Yemen S Dwyer ST. Compartmentalized connexin 43 s-nitrosylation/denitrosylation regulates heterocellular communication in the vessel wall. Arterioscler Thromb Vasc Biol. (2011) 31:399–407. 10.1161/ATVBAHA.110.215939 21071693 PMC3056333

[22] Straub AC Johnstone SR Heberlein KR Rizzo MJ Best AK Boitano S et al. Site-specific connexin phosphorylation is associated with reduced heterocellular communication between smooth muscle and endothelium. J Vasc Res. (2010) 47:277–86. 10.1159/000265562 20016202 PMC2895757

[23] Braun M Pietsch P Schror K Baumann G Felix SB. Cellular adhesion molecules on vascular smooth muscle cells. Cardiovasc Res. (1999) 41:395–401. 10.1016/S0008-6363(98)00302-2 10341839

[24] Tanaka H Sukhova GK Swanson SJ Clinton SK Ganz P Cybulsky MI et al. Sustained activation of vascular cells and leukocytes in the rabbit aorta after balloon injury. Circulation (1993) 88:1788–803. 10.1161/01.CIR.88.4.1788 7691431

[25] Yoshida T Owens GK. Molecular determinants of vascular smooth muscle cell diversity. Circ Res. (2005) 96:280–91. 10.1161/01.RES.0000155951.62152.2e 15718508

[26] Liu H Kennard S Lilly B. NOTCH3 expression is induced in mural cells through an autoregulatory loop that requires endothelial-expressed JAGGED1. Circ Res. (2009) 104:466–75. 10.1161/CIRCRESAHA.108.184846 19150886 PMC2747310

[27] Kuijper S Turner CJ Adams RH. Regulation of angiogenesis by Eph-ephrin interactions. Trends Cardiovasc Med. (2007) 17:145–51. 10.1016/j.tcm.2007.03.003 17574121

[28] Pitulescu ME Adams RH. Regulation of signaling interactions and receptor endocytosis in growing blood vessels. Cell Adh Migr. (2014) 8:366–77. 10.4161/19336918.2014.970010 25482636 PMC4594521

[29] Foo SS Turner CJ Adams S Compagni A Aubyn D Kogata N. Ephrin-B2 controls cell motility and adhesion during blood-vessel-wall assembly. Cell (2006) 124:161–73. 10.1016/j.cell.2005.10.034 16413489

[30] Sakamoto A Sugamoto Y Tokunaga Y Yoshimuta T Hayashi K Konno T. Expression profiling of the ephrin (EFN) and Eph receptor (EPH) family of genes in atherosclerosis-related human cells. J Int Med Res. (2011) 39:522–7. 10.1177/147323001103900220 21672356

[31] Sakamoto A Ishibashi-Ueda H Sugamoto Y Higashikata T Miyamoto S Kawashiri MA. Expression and function of ephrin-B1 and its cognate receptor EphB2 in human atherosclerosis: from an aspect of chemotaxis. Clin Sci. (2008) 114:643–50. 10.1042/CS20070339 18092944

[32] Barquilla A Pasquale EB. Eph receptors and ephrins: therapeutic opportunities. Annu Rev Pharmacol Toxicol. (2015) 55:465–87. 10.1146/annurev-pharmtox-011112-140226 25292427 PMC4388660

[33] Dora KA. Cell-cell communication in the vessel wall. Vasc. Med. (2001) 6:43–50. 10.1177/1358836X0100600108 11358160

[34] Yu J Zhang Y Zhang X Rudic RD Bauer PM Altieri DC Sessa WC. Endothelium derived nitric oxide synthase negatively regulates the PDGF–survivin pathway during flow-dependent vascular remodeling. PLoS ONE (2012) 7:e31495. 10.1371/journal.pone.0031495 22355372 PMC3280303

[35] Sandow SL Bramich NJ Bandi HP Rummery NM Hill CE. Structure, function, and endothelium-derived hyperpolarizing factor in the caudal artery of the SHR and WKY rat. Arterioscler Thromb Vasc Biol. (2003) 23:822–8. 10.1161/01.ATV.0000067425.06019.D7 12649082

[36] Tang EH and Vanhoutte PM. Gap junction inhibitors reduce endothelium-dependent contractions in the aorta of spontaneously hypertensive rats. J Pharmacol Exp Ther. (2008) 327:148–53. 10.1124/jpet.108.140046 18632992

[37] Griffith TM. Endothelium-dependent smooth muscle hyperpolarization: do gap junctions provide a unifying hypothesis? Br J Pharmacol. (2004) 141:881–903. 10.1038/sj.bjp.0705698 15028638 PMC1574270

[38] Griffith TM Chaytor AT Edwards DH. The obligatory link: role of gap junctional communication in endothelium-dependent smooth muscle hyperpolarization. Pharmacol Res. (2004) 49:551–64. 10.1016/j.phrs.2003.11.014 15026033

[39] Lutter S Xie S Tatin F Makinen T. Smooth muscle-endothelial cell communication activates Reelin signaling and regulates lymphatic vessel formation. J Cell Biol. (2012) 197:837–49. 10.1083/jcb.201110132 22665518 PMC3373399

[40] Wagenseil JE Mecham RP. Vascular extracellular matrix and arterial mechanics. Physiol Rev. (2009) 89:957–89. 10.1152/physrev.00041.2008 19584318 PMC2775470

[41] Davis GE Senger DR. Endothelial extracellular matrix: biosynthesis, remodeling, and functions during vascular morphogenesis and neovessel stabilization. Circ Res. (2005) 97:1093–107. 10.1161/01.RES.0000191547.64391.e3 16306453

[42] Yu PJ Ferrari G Pirelli L Gulkarov I Galloway AC Mignatti P et al. Vascular injury and modulation of MAPKs: a targeted approach to therapy of restenosis. Cell. signal. (2007) 19:1359–71. 10.1016/j.cellsig.2007.03.002 17448633

[43] Hafiane A Daskalopoulou SS. Extracellular vesicles characteristics and emerging roles in atherosclerotic cardiovascular disease. Metabolism (2018) 85:213–22. 10.1016/j.metabol.2018.04.008 29727628

[44] Hutcheson JD Aikawa E. Extracellular vesicles in cardiovascular homeostasis and disease. Curr Opin Cardiol. (2018) 33:290–7. 10.1097/HCO.0000000000000510 29465447 PMC5895489

[45] Chistiakov DA Orekhov AN Bobryshev YV. Extracellular vesicles and atherosclerotic disease. Cell Mol Life Sci. (2015) 72:2697–708. 10.1007/s00018-015-1906-2 25894694 PMC11113133

[46] Holnthoner W Bonstingl C Hromada C Muehleder S Zipperle J Stojkovic S. Endothelial cell-derived extracellular vesicles size-dependently exert procoagulant activity detected by thromboelastometry. Sci Rep. (2017) 7:3707. 10.1038/s41598-017-03159-0 28623360 PMC5473891

[47] Kapustin AN Schoppet M Schurgers LJ Reynolds JL McNair R Heiss A. Prothrombin loading of vascular smooth muscle cell-derived exosomes regulates coagulation and calcification. Arterioscler Thromb Vasc Biol. (2017) 37:e22–e32. 10.1161/ATVBAHA.116.308886 28104608

[48] Sitikov AS. Antisense RNAs as envoys in intercellular communication: 20 years later. Biochemistry (Mosc). (2012) 77:1478–86. 10.1134/S0006297912130068 23379523

[49] Feinberg MW Moore KJ. MicroRNA regulation of atherosclerosis. Circ Res. (2016) 118:703–20. 10.1161/CIRCRESAHA.115.306300 26892968 PMC4762069

[50] Hergenreider E Heydt S Treguer K Boettger T Horrevoets AJ Zeiher AM. Atheroprotective communication between endothelial cells and smooth muscle cells through miRNAs. Nat Cell Biol. (2012) 14:249–56. 10.1038/ncb2441 22327366

[51] Zhou J Li YS Nguyen P Wang KC Weiss A Kuo YC. Regulation of vascular smooth muscle cell turnover by endothelial cell-secreted microRNA-126: role of shear stress. Circ Res. (2013) 113:40–51. 10.1161/CIRCRESAHA.113.280883 23603512 PMC3772783

[52] Baker AB Ettenson DS Jonas M Nugent MA Iozzo RV Edelman ER. Endothelial cells provide feedback control for vascular remodeling through a mechanosensitive autocrine TGF-beta signaling pathway. Circ Res. (2008) 103:289–97. 10.1161/CIRCRESAHA.108.179465 18583708 PMC2766078

[53] Qi YX Jiang J Jiang XH Wang XD Ji SY Han Y. PDGF-BB and TGF-{beta}1 on cross-talk between endothelial and smooth muscle cells in vascular remodeling induced by low shear stress. Proc Natl Acad Sci USA. (2011) 108:1908–13. 10.1073/pnas.1019219108 21245329 PMC3033274

[54] Kang Y Kim J Anderson JP Wu J Gleim SR Kundu RK. Apelin-APJ signaling is a critical regulator of endothelial MEF2 activation in cardiovascular development. Circ Res. (2013) 113:22–31. 10.1161/CIRCRESAHA.113.301324 23603510 PMC3739451

[55] Che J Okigaki M Takahashi T Katsume A Adachi Y Yamaguchi S. Endothelial FGF receptor signaling accelerates atherosclerosis. Am J Physiol Heart Circ Physiol. (2011) 300:H154–61. 10.1152/ajpheart.00075.2010 20952669

[56] Yang J Sato K Aprahamian T Brown NJ Hutcheson J Bialik A. Endothelial overexpression of Fas ligand decreases atherosclerosis in apolipoprotein E-deficient mice. Arterioscler Thromb Vasc Biol. (2004) 24:1466–73. 10.1161/01.ATV.0000134402.94963.2f 15178561

[57] Zhang D Chen Y Xie X Liu J Wang Q Kong W et al. Homocysteine activates vascular smooth muscle cells by DNA demethylation of platelet-derived growth factor in endothelial cells. J Mol Cell Cardiol. (2012) 53:487–96. 10.1016/j.yjmcc.2012.07.010 22867875

[58] Breen DM Chan KK Dhaliwall JK Ward MR Al Koudsi N Lam L. Insulin increases reendothelialization and inhibits cell migration and neointimal growth after arterial injury. Arterioscler Thromb Vasc Biol. (2009) 29:1060–6. 10.1161/ATVBAHA.109.185447 19359661

[59] Fuchs AT Kuehnl A Pelisek J Rolland PH Mekkaoui C Netz H Nikol S. Inhibition of restenosis formation without compromising reendothelialization as a potential solution to thrombosis following angioplasty? Endothelium (2008) 15:85–92. 10.1080/10623320802092484 18568948

[60] Kang DH Lee DJ Kim J Lee JY Kim HW Kwon K. Vascular injury involves the overoxidation of peroxiredoxin type II and is recovered by the peroxiredoxin activity mimetic that induces reendothelialization. Circulation (2013) 128:834–44. 10.1161/CIRCULATIONAHA.113.001725 23820076 PMC5479486

[61] Kim HJ Kim JY Lee SJ Kim HJ Oh CJ Choi YK. alpha-Lipoic acid prevents neointimal hyperplasia *via* induction of p38 mitogen-activated protein kinase/Nur77-mediated apoptosis of vascular smooth muscle cells and accelerates postinjury reendothelialization. Arterioscler Thromb Vasc Biol. (2010) 30:2164–72. 10.1161/ATVBAHA.110.212308 20829507

[62] Matthijs Blankesteijn W Hermans KC Wnt signaling in atherosclerosis. Eur J Pharmacol. (2015) 763:122–30. 10.1016/j.ejphar.2015.05.023 25987418

[63] Abou Ziki MD and Mani A Wnt signaling, a novel pathway regulating blood pressure? State of the art review. Atherosclerosis (2017) 262:171–8. 10.1016/j.atherosclerosis.2017.05.001 28522145 PMC5508596

[64] Marinou K Christodoulides C Antoniades C Koutsilieris M. Wnt signaling in cardiovascular physiology. Trends Endocrinol Metab. (2012) 23:628–36. 10.1016/j.tem.2012.06.001. 22902904

[65] Vikram A Kim YR Kumar S Naqvi A Hoffman TA Kumar A. Canonical Wnt signaling induces vascular endothelial dysfunction *via* p66Shc-regulated reactive oxygen species. Arterioscler Thromb Vasc Biol. (2014) 34:2301–9. 10.1161/ATVBAHA.114.304338 25147340 PMC6069972

[66] Bhatt PM Malgor R. Wnt5a: a player in the pathogenesis of atherosclerosis and other inflammatory disorders. Atherosclerosis (2014) 237:155–62. 10.1016/j.atherosclerosis.2014.08.027 25240110 PMC4252768

[67] Tsaousi A Williams H Lyon CA Taylor V Swain A Johnson JL et al. Wnt4/beta-catenin signaling induces VSMC proliferation and is associated with intimal thickening. Circ Res. (2011) 108:427–36. 10.1161/CIRCRESAHA.110.233999 21193738

[68] Wang X Adhikari N Li Q Guan Z Hall JL The role of [beta]-transducin repeat-containing protein ([beta]-TrCP) in the regulation of NF-[kappa]B in vascular smooth muscle cells. Arterioscler Thromb Vasc Biol. (2004) 24:85–90. 10.1161/01.ATV.0000104012.40720.c4 14592850

[69] Korn C Scholz B Hu J Srivastava K Wojtarowicz J Arnsperger T. Endothelial cell-derived non-canonical Wnt ligands control vascular pruning in angiogenesis. Development (2014) 141:1757–66. 10.1242/dev.104422 24715464

[70] Scholz B Korn C Wojtarowicz J Mogler C Augustin I Boutros M Endothelial RSPO3 controls vascular stability and pruning through non-canonical WNT/Ca^2+^/NFAT signaling. Dev Cell (2016) 36:79–93. 10.1016/j.devcel.2015.12.015 26766444

[71] Gay A Towler DA. Wnt signaling in cardiovascular disease: opportunities and challenges. Curr Opin Lipidol. (2017) 28:387–96. 10.1097/MOL.0000000000000445 28723729 PMC5773247

[72] Feil S Fehrenbacher B Lukowski R Essmann F Schulze-Osthoff K Schaller M et al. Transdifferentiation of vascular smooth muscle cells to macrophage-like cells during atherogenesis. Circ Res. (2014) 115:662–7. 10.1161/CIRCRESAHA.115.304634 25070003

[73] Coll-Bonfill N Musri MM Ivo V Barbera JA Tura-Ceide O. Transdifferentiation of endothelial cells to smooth muscle cells play an important role in vascular remodelling. Am J Stem Cells (2015) 4:13–21. 25973327 PMC4396157

[74] Nikitopoulou I Orfanos SE Kotanidou A Maltabe V Manitsopoulos N Karras P. Vascular endothelial-cadherin downregulation as a feature of endothelial transdifferentiation in monocrotaline-induced pulmonary hypertension. Am J Physiol Lung Cell Mol Physiol. (2016) 311:L352–63. 10.1152/ajplung.00156.2014 27233997

[75] Zhu P Huang L Ge X Yan F Wu R Ao Q. Transdifferentiation of pulmonary arteriolar endothelial cells into smooth muscle-like cells regulated by myocardin involved in hypoxia-induced pulmonary vascular remodelling. Int J Exp Pathol. (2006) 87:463–74. 10.1111/j.1365-2613.2006.00503.x 17222214 PMC2517388

[76] Davignon J Ganz P. Role of endothelial dysfunction in atherosclerosis. Circulation (2004) 109:III27–32. 10.1161/01.CIR.0000131515.03336.f8 15198963

[77] Jia LX Zhang WM Li TT Liu Y Piao CM Ma YC. ER stress dependent microparticles derived from smooth muscle cells promote endothelial dysfunction during thoracic aortic aneurysm and dissection. Clin Sci. (2017) 131:1287–99. 10.1042/CS20170252 28468950 PMC5461939

[78] Frismantiene A Philippova M Erne P Resink TJ. Smooth muscle cell-driven vascular diseases and molecular mechanisms of VSMC plasticity. Cell signal. (2018) 52:48–64. 10.1016/j.cellsig.2018.08.019 30172025

[79] Joutel A Corpechot C Ducros A Vahedi K Chabriat H Mouton P. Notch3 mutations in CADASIL, a hereditary adult-onset condition causing stroke and dementia. Nature (1996) 383:707–10. 10.1038/383707a0 8878478

[80] Peters N Freilinger T Opherk C Pfefferkorn T Dichgans M. Enhanced L-arginine-induced vasoreactivity suggests endothelial dysfunction in CADASIL. J Neurol. (2008) 255:1203–8. 10.1007/s00415-008-0876-9 18537053

[81] Belin de Chantemele EJ Retailleau K Pinaud F Vessieres E Bocquet A Guihot AL Notch3 is a major regulator of vascular tone in cerebral and tail resistance arteries. Arterioscler Thromb Vasc Biol. (2008) 28:2216–24. 10.1161/ATVBAHA.108.171751 18818417 PMC2658748

[82] Arboleda-Velasquez JF Manent J Lee JH Tikka S Ospina C Vanderburg CR. Hypomorphic Notch 3 alleles link Notch signaling to ischemic cerebral small-vessel disease. Proc Natl Acad Sci USA. (2011) 108:E128–35. 10.1073/pnas.1101964108 21555590 PMC3102344

[83] Neptune ER Frischmeyer PA Arking DE Myers L Bunton TE Gayraud B. Dysregulation of TGF-beta activation contributes to pathogenesis in Marfan syndrome. Nat Genet. (2003) 33:407–11. 10.1038/ng1116 12598898

[84] Doyle JJ Gerber EE Dietz HC. Matrix-dependent perturbation of TGFbeta signaling and disease. FEBS Lett. (2012) 586:2003–15. 10.1016/j.febslet.2012.05.027 22641039 PMC3426037

[85] Syyong HT Chung AW Yang HH van Breemen C. Dysfunction of endothelial and smooth muscle cells in small arteries of a mouse model of Marfan syndrome. Br J Pharmacol. (2009) 158:1597–608. 10.1111/j.1476-5381.2009.00439.x 19814726 PMC2795226

[86] McAllister KA Grogg KM Johnson DW Gallione CJ Baldwin MA Jackson CE. Endoglin, a TGF-beta binding protein of endothelial cells, is the gene for hereditary haemorrhagic telangiectasia type 1. Nat Genet. (1994) 8:345–51. 10.1038/ng1294-345 7894484

[87] Bernabeu C Conley BA Vary CP. Novel biochemical pathways of endoglin in vascular cell physiology. J Cell Biochem. (2007) 102:1375–88. 10.1002/jcb.21594 17975795 PMC2199238

[88] Lebrin F Mummery CL. Endoglin-mediated vascular remodeling: mechanisms underlying hereditary hemorrhagic telangiectasia. Trends Cardiovasc Med. (2008) 18:25–32. 10.1016/j.tcm.2007.11.003 18206806

[89] Whitehead KJ Smith MC Li DY. Arteriovenous malformations and other vascular malformation syndromes. Cold Spring Harb Perspect Med. (2013) 3:a006635. 10.1101/cshperspect.a006635 23125071 PMC3552339

[90] Johnson DW Berg JN Baldwin MA Gallione CJ Marondel I Yoon SJ. Mutations in the activin receptor-like kinase 1 gene in hereditary haemorrhagic telangiectasia type 2. Nat Genet. (1996) 13:189–95. 10.1038/ng0696-189 8640225

[91] Mancini ML Terzic A Conley BA Oxburgh LH Nicola T Vary CP. Endoglin plays distinct roles in vascular smooth muscle cell recruitment and regulation of arteriovenous identity during angiogenesis. Dev Dyn. (2009) 238:2479–93. 10.1002/dvdy.22066 19705428 PMC2947792

[92] Toporsian M Gros R Kabir MG Vera S Govindaraju K Eidelman DH. A role for endoglin in coupling eNOS activity and regulating vascular tone revealed in hereditary hemorrhagic telangiectasia. Circ Res. (2005) 96:684–92. 10.1161/01.RES.0000159936.38601.22 15718503

[93] de Zeeuw P Wong BW Carmeliet P Metabolic adaptations in diabetic endothelial cells. Circ J. (2015) 79:934–41. 10.1253/circj.CJ-15-0230 25787231

[94] Missiaen R Morales-Rodriguez F Eelen G Carmeliet P. Targeting endothelial metabolism for anti-angiogenesis therapy: a pharmacological perspective. Vasc Pharmacol. (2017) 90:8–18. 10.1016/j.vph.2017.01.001 28082117

[95] Tabit CE Chung WB Hamburg NM Vita JA. Endothelial dysfunction in diabetes mellitus: molecular mechanisms and clinical implications. Rev Endocr Metab Disord. (2010) 11:61–74. 10.1007/s11154-010-9134-4 20186491 PMC2882637

[96] Roberts AC Porter KE. Cellular and molecular mechanisms of endothelial dysfunction in diabetes. Diab Vasc Dis Res. (2013) 10:472–82. 10.1177/1479164113500680 24002671

[97] Widlansky ME Gokce N Keaney JF Jr Vita JA. The clinical implications of endothelial dysfunction. J Am Coll Cardiol. (2003) 42:1149–60. 10.1016/S0735-1097(03)00994-X 14522472

[98] Ramasamy SK Kusumbe AP Adams RH. Regulation of tissue morphogenesis by endothelial cell-derived signals. Trends Cell Biol. (2015) 25:148–57. 10.1016/j.tcb.2014.11.007 25529933 PMC4943524

[99] Eelen G de Zeeuw P Treps L Harjes U Wong BW Carmeliet P. Endothelial cell metabolism. Physiol Rev. (2018) 98:3–58. 10.1152/physrev.00001.2017 29167330 PMC5866357

[100] Rohlenova K Veys K Miranda-Santos I De Bock K Carmeliet P. Endothelial cell metabolism in health and disease. Trends Cell Biol. (2018) 28:224–36. 10.1016/j.tcb.2017.10.010 29153487

[101] Navaratna D Guo S Arai K Lo EH. Mechanisms and targets for angiogenic therapy after stroke. Cell Adh Migr. (2009) 3:216–23. 10.4161/cam.3.2.8396 19363301 PMC2679890

[102] Baratchi S Khoshmanesh K Woodman OL Potocnik S Peter K McIntyre P. Molecular sensors of blood flow in endothelial cells. Trends Mol Med. (2017) 23:850–68. 10.1016/j.molmed.2017.07.007 28811171

[103] Li M Qian M Xu J. Vascular endothelial regulation of obesity-associated insulin resistance. Front Cardiovasc Med. (2017) 4:51. 10.3389/fcvm.2017.00051 28848738 PMC5552760

[104] Dejana E Tournier-Lasserve E Weinstein BM. The control of vascular integrity by endothelial cell junctions: molecular basis and pathological implications. Dev Cell (2009) 16:209–21. 10.1016/j.devcel.2009.01.004 19217423

[105] Giannotta M Trani M Dejana E. VE-cadherin and endothelial adherens junctions: active guardians of vascular integrity. Dev Cell (2013) 26:441–54. 10.1016/j.devcel.2013.08.020 24044891

[106] Cerutti C Ridley AJ. Endothelial cell-cell adhesion and signaling. Exp Cell Res. (2017) 358:31–8. 10.1016/j.yexcr.2017.06.003 28602626 PMC5700119

[107] Abraham S Yeo M Montero-Balaguer M Paterson H Dejana E Marshall CJ et al. VE-Cadherin-mediated cell-cell interaction suppresses sprouting *via* signaling to MLC2 phosphorylation. Curr Biol. (2009) 19:668–74. 10.1016/j.cub.2009.02.057 19345098

[108] Jacot JG Wong JY. Endothelial injury induces vascular smooth muscle cell proliferation in highly localized regions of a direct contact co-culture system. Cell Biochem Biophys. (2008) 52:37–46. 10.1007/s12013-008-9023-6 18766304 PMC2770599

[109] Mudau M Genis A Lochner A Strijdom H. Endothelial dysfunction: the early predictor of atherosclerosis. Cardiovasc J Africa (2012) 23:222–31. 10.5830/CVJA-2011-068 22614668 PMC3721957

[110] Xi D Zhao J Lai W Guo Z. Systematic analysis of the molecular mechanism underlying atherosclerosis using a text mining approach. Hum Genomics (2016) 10:14. 10.1186/s40246-016-0075-1 27251057 PMC4890502

[111] Bochaton-Piallat ML Back M. Novel concepts for the role of smooth muscle cells in vascular disease: towards a new smooth muscle cell classification. Cardiovasc Res. (2018) 114:477–80. 10.1093/cvr/cvy031 29408963

[112] Parks BW Lusis AJ. Macrophage accumulation in atherosclerosis. N Engl J Med. (2013) 369:2352–3. 10.1056/NEJMcibr1312709 24328470 PMC3934498

[113] Nachman RL Rafii S. Platelets, petechiae, and preservation of the vascular wall. N Engl J Med. (2008) 359:1261–70. 10.1056/NEJMra0800887 18799560 PMC2935201

[114] Chen YC Peter K. Determining the characteristics of human atherosclerosis: a difficult but indispensable task providing the direction and proof of concept for pioneering atherosclerosis research in animal models. Atherosclerosis (2015) 241:595–6. 10.1016/j.atherosclerosis.2015.06.009 26115071

[115] Kaplan JR Adams MR Clarkson TB Manuck SB Shively CA Williams JK. Psychosocial factors, sex differences, and atherosclerosis: lessons from animal models. Psychosom Med. (1996) 58:598–611. 10.1097/00006842-199611000-00008 8948008

[116] Vesselinovitch D Wissler RW. Comparison of primates and rabbits as animal models in experimental atherosclerosis. Adv Exp Med Biol. (1977) 82:614–22. 411342 10.1007/978-1-4613-4220-5_131

[117] Getz GS Reardon CA. Animal models of atherosclerosis. Arterioscler Thromb Vasc Biol. (2012) 32:1104–15. 10.1161/ATVBAHA.111.237693 22383700 PMC3331926

[118] Yahagi K Davis HR Arbustini E Virmani R. Sex differences in coronary artery disease: pathological observations. Atherosclerosis (2015) 239:260–7. 10.1016/j.atherosclerosis.2015.01.017 25634157

[119] Franconi F Rosano G Basili S Montella A Campesi I. Human cells involved in atherosclerosis have a sex. Int J Cardiol. (2017) 228:983–1001. 10.1016/j.ijcard.2016.11.118 27915217

